# Distribution and current infection status of *Biomphalaria straminea* in Hong Kong

**DOI:** 10.1186/s13071-017-2285-3

**Published:** 2017-07-25

**Authors:** Xin Zeng, Wing Chung Yiu, Kwan Ho Cheung, Ho Yin Yip, Wenyan Nong, Ping He, Dongjuan Yuan, David Rollinson, Jian-Wen Qiu, Ming Chiu Fung, Zhongdao Wu, Jerome Ho Lam Hui

**Affiliations:** 1 School of Life Science, State Key Laboratory of Agrobiotechnology, The Chinese University of Hong Kong, Hong Kong, Special Administrative Region, People’s Republic of China; 2 Department of Parasitology, Zhongshan School of Medicine, Sun Yat-sen University, Guangzhou, Guangdong Province People’s Republic of China; 3 Key Laboratory of Tropical Disease Control (SYSU), Ministry of Education, Guangzhou, Guangdong Province People’s Republic of China; 4 Wolfson Wellcome Biomedical Laboratories, Department of Life Sciences, Natural History Museum, Cromwell Road, London, SW7 5BD UK; 5 Department of Biology, Hong Kong Baptist University, Hong Kong, Special Administrative Region, People’s Republic of China

**Keywords:** Schistosomiasis, *Schistosoma mansoni*, *Biomphalaria straminea*, Hong Kong, China

## Abstract

**Background:**

Schistosomiasis, also generally known as snail fever, is a parasitic disease caused by trematode flatworms of the genus *Schistosoma*. In Hong Kong and mainland China, the freshwater snail *Biomphalaria straminea* has been introduced and has the potential to transmit intestinal schistosomiasis caused by *S. mansoni*, a parasite of man which has a wide distribution in Africa and parts of the New World, especially Brazil. The first identification of *B. straminea* in Hong Kong dates back to the 1970s, and its geographical distribution, phylogenetic relationships, and infection status have not been updated for more than 30 years. Thus, this study aims to reveal the distribution and current infection status of *B. straminea* in contemporary Hong Kong.

**Methods:**

Snails were collected from different parts of Hong Kong from July 2016 to January 2017. Both anatomical and molecular methods were applied to identify *B. straminea*. Cytochrome *c* oxidase subunit 1 (*cox*1), internal transcribed spacer 1 (ITS1), 5.8S rDNA, internal transcribed spacer 2 (ITS2), and 16S ribosomal DNA (rDNA) were sequenced from individual snails and analyzed. To detect the presence of *S. mansoni*, both biopsy and PCR analyses were carried out.

**Results:**

Using both anatomical and molecular analyses, this study demonstrated the existence of black- and red-coloured shell *B. straminea* in different districts in the New Territories in Hong Kong, including places close to the mainland China border. None of the *B. straminea* (*n* = 87) investigated were found to be infected with *S. mansoni* when tested by biopsy and PCR. The Hong Kong *B. straminea* are genetically indistinguishable, based on the chosen molecular markers (*cox*1, ITS1-5.8S-ITS2, and 16S rDNA), and are similar to those obtained in mainland China and South America.

**Conclusion:**

*Biomphalaria straminea* is now well established in freshwater habitats in Hong Kong. No evidence of infection with *S. mansoni* has been found. Surveillance should be continued to monitor and better understand this schistosomiasis intermediate host in mainland China and Hong Kong.

**Electronic supplementary material:**

The online version of this article (doi:10.1186/s13071-017-2285-3) contains supplementary material, which is available to authorized users.

## Background

With an estimate of almost 240 million people infected worldwide, schistosomiasis is considered by the World Health Organization as the second most prevalent parasitic disease after malaria. This disease remains a global health problem resulting in economic and social burdens [1]. One of the most widespread of the human-infecting species is *Schistosoma mansoni*, which is estimated to infect more than 80 million people globally. Intestinal schistosomiasis caused by *S. mansoni* occurs in Africa, Madagascar, the Middle East, the Caribbean, Brazil, Venezuela and Suriname.

Infections with *S. mansoni* in humans are initiated by the release of cercariae by various species of freshwater snails of the genus *Biomphalaria*; cercariae penetrate the skin of people when exposed in water. Given the parasite life-cycle and a combination of other factors, including poor public health education, water quality, sanitation and natural disasters (such as flooding that may spread the snails), the number of people infected with *S. mansoni* is especially high in socioeconomic underdeveloped countries [1]. Intestinal schistosomiasis caused by *S. mansoni* is not currently endemic in Hong Kong and mainland China, while another species *Schistosoma japonicum* transmitted by the prosobranch snails *Oncomelania* spp., has been the subject of intense control measures in China. According to the Law of Communicable Diseases Prevention and Control in China, schistosomiasis is currently listed in the category B of notifiable diseases to be reported. In 2011, there were a total of 4483 cases of schistosomiasis caused by *Schistosoma japonicum* reported to the Ministry of Health nationwide in China. Recently, imported cases of schistosomiasis caused by *Schistosoma mansoni* have been reported to be increasing in China [2].

To date, there are 34 described species of *Biomphalaria* snails, 18 of which have been demonstrated to be infected by *S. mansoni,* either naturally or experimentally [3]. Different geographical locations are dominated by different species of *Biomphalaria*, for instance, *B. pfeifferi*, *B. alexandrina* and *B. sudanica* serve as the main intermediate hosts of *S. mansoni* in Africa, Madagascar and the Middle East, while *B. glabrata* dominates in the Neotropics. In some areas, other species of *Biomphalaria* such as *B. straminea*, *B. tenagophila*, *B. choanomphala*, *B. camerunensis* and *B. stanleyi*, have been shown to play important roles in transmitting schistosomiasis [3–6]. Transmission of imported schistosomiasis will not occur in places without the host snails [7, 8], and hence, understanding the biology and distribution of the intermediate hosts contributes to the prevention and control of schistosomiasis.

Globalization and traveling are two important factors that aid the spread of schistosomiasis. For example, urogenital schistosomiasis has been detected in different parts of Europe, primarily in travellers returning from endemic areas where they have been exposed to water containing cercariae of *S. haematobium* [9–12]. Recently, *S. haematobium* was introduced into the island of Corsica and became established in the local populations of *Bulinus truncatus*. This resulted in a number of local people and tourists becoming infected with urogenital schistosomiasis [9–11]. Previous studies have revealed that the schistosomiasis prevalence rate in sub-Saharan African immigrants during 2000–2009 in Spain was ~5.9% (*n* = 317), while the Hospital for Tropical Diseases in the United Kingdom diagnosed 1020 schistosomiasis cases in travellers returning from Africa between 1997 to 2012; *S. haematobium* was the predominant species in those with schistosomiasis confirmed by microscopy (74.2%, 204/252) [13, 14].

In 2012, the Guangdong Provincial Center for Disease Control and Prevention (CDC) performed a survey and recorded *B. straminea* in the water areas of Shenzhen and most of the rivers in the Lo Wu area (which is the border between Hong Kong and mainland China). A recent survey has further supported that *B. straminea* and *B. kuhniana* (the latter is thought to be incompatible with *S. mansoni*) can be found in different regions in the southern part of China - Guangdong [15], suggesting a surveillance-response system is required. Trading and travel between different places in the globe, and especially between Guangdong and Hong Kong, have been increasing rapidly in the last decades after the handover. Many businessmen, workers and tourists may visit schistosomiasis-endemic areas and become infected; it is the movement of people that provides the opportunity for the parasite to be introduced into a new area. Even when good sanitation exists, it is possible that the local water could become contaminated by bathing or defecation. If a compatible snail host is present, there is a chance of transmission becoming established.

Indeed, back in 1970s, the occurrence of *B. straminea* was first reported at Lam Tsuen valley in Hong Kong [16], and snails have since been identified at different places in Hong Kong and Guangdong Province [17–19]. Molecular (mainly allozyme electrophoresis) and field studies in those days suggested that there could have been multiple introductions of this species to Hong Kong via imported tropical aquarium plants or fish from South America [18, 19]. From 1979 to 2011, imported schistosomiasis cases (total 426) continued to be reported in China, and ~69% (294) of them were reported after 2000 [20]. The snail *B. straminea* can be relatively fast spreading (i.e. lays as many as ~70 eggs/snail/day), and is tolerant of drought for more than 30 days [21]. These features give it the potential for dispersal between Hong Kong and other areas in China, such as by attaching to floating items and transfer from one city to another during storms (e.g. [15, 16, 19]). However, there has not been any systematic monitoring programme or vigilance for *B. straminea*’s migration and current status in Hong Kong.

It is evident that the situation regarding the current geographical distribution, evolutionary history and infection of *B. straminea* by *S. mansoni*, is poorly known in contemporary Hong Kong. The present study carried out from July 2016 to January 2017 revealed that at least eight locations in the New Territories in Hong Kong contained *B. straminea*. Moreover, a variant strain of *B. straminea* with red-coloured shells was discovered. Although the snails collected in the field were found not to be infected with *S. mansoni*, this study confirms the continued existence of Neotropical schistosomiasis-transmitting snails in contemporary Hong Kong, and warrants the monitoring and understanding of the biology of this intermediate host between the borders of mainland China and Hong Kong.

## Methods

### Specimen collection

Hong Kong is a place with a great variety of habitats, including grasslands, shrublands, woodlands, hilltops, streams, ponds, wetlands, agricultural fields and urban areas. The climate is sub-tropical, and there are usually heavy rainfalls and tropical cyclones from June to October. Snails with shell morphology similar to *B. straminea* were collected from rivers, ditches and reservoirs in different parts of Hong Kong from July 2016 to January 2017. For safety measures, researchers wore rubber gloves and Wellington boots to prevent direct skin contact with water and snails. Specimens were labeled with collection locality, GPS coordinates, date and collector, and transported to the laboratory for storage in 95% ethanol; these data are summarized in Table 1.

**Table 1:** Field sampling sites for snails *Biomphalaria straminea* in Hong Kong

Sampling site	District	GPS coordinates	*B. straminea*
Lo Wu Border (LW)	New Territories	22.526486, 114.112326	Yes
Ping Che Road (PCL)	New Territories	22.516650, 114.166232	Yes
Shek Sheung River(SSH)	New Territories	22.505979, 114.118531	Yes
Ng Tung River (NgT)	New Territories	22.507454, 114.135875	Yes
Shan Pui River 1 (SB)	New Territories	22.455540, 114.031890	Yes
Kam Po Road (JP)	New Territories	22.433702, 114.067210	Yes
Fu Hang Road (HKL)	New Territories	22.414986, 113.984772	Yes
Sam Long Apartments (SG)	New Territories	22.333522, 114.239565	Yes
Little Hawaii Trail	New Territories	22.331859, 114.247215	No
Ta Shek Wu	New Territories	22.462304, 114.106114	No
Tan Chuk Hang	New Territories	22.512544, 114.177685	No
Chinese University of Hong Kong	New Territories	22.416144, 114.207924	No
Upper Shing Mun Reservoir	New Territories	22.392784, 114.149916	No
Kowloon Reservoir	New Territories	22.351072, 114.153883	No
Ho Chung	New Territories	22.354735, 114.246225	No
Lam Uk Tsuen	New Territories	22.451903, 114.027649	No
Fairview Park	New Territories	22.472910, 114.050549	No
Shan Pui River 2	New Territories	22.469330, 114.030305	No
Lee Uk Tsuen	New Territories	22.537282, 114.150019	No
Tung Chung River	Lantau Island	22.276882, 113.931202	No
Wong Lung Hang Road	Lantau Island	22.279049, 113.941154	No
Hong Kong University	Hong Kong Island	22.282796, 114.138440	No
Hong Kong Park	Hong Kong Island	22.277658, 114.162057	No
Aldrich Bay Park	Hong Kong Island	22.282800, 114.224999	No
Aberdeen Country Park	Hong Kong Island	22.282924, 114.225162	No
Wong Nai Chung Reservoir Park	Hong Kong Island	22.282924, 114.225162	No
Tai Tam Tuk Reservoir	Hong Kong Island	22.282924, 114.225162	No

### Anatomical and molecular identification of *B. straminea*

Snails collected from each location were dissected for anatomical identification under a microscope [22]. In brief, shells of snails were carefully removed with forceps and the number of prostate diverticula were counted under the microscope as previously described [22]. Genomic DNA was further extracted from ~10–30 mg foot tissue of snails examined above using QIAamp DNA Mini Kit (Qiagen, Hilden, Germany). DNA sequencing of individual cytochrome *c* oxidase subunit 1 (*cox*1) [23], internal transcribed spacer 1 (ITS1), 5.8S rDNA, and internal transcribed spacer 2 (ITS2), and 16S ribosomal DNA (rDNA) was carried out as previously described [24, 25]. All 3 markers were amplified under the same PCR temperature profile: an initial 5 min denaturation step at 94 °C followed by 30 cycles (iterations of 50 s at 94 °C, 50 s at 55 °C and 50 s at 72 °C) plus a final 10 min extension at 72 °C. Gel electrophoreses were performed to confirm successful amplification of the desired fragment of the target size. PCR products were purified using the QIAquick PCR Product Purification Kit (Qiagen, Hilden, Germany). Sequences of purified PCR products were obtained from both directions using the same primer pair for PCR by cycle sequencing in a commercial laboratory (BGI Tech Solutions (Hong Kong) Co., Ltd., Beijing, China).

### Phylogenetic analyses of *B. straminea*

The *cox*1, ITS1-5.8S-ITS2 and 16S rDNA sequences obtained above and those available at the NCBI databases (Tables 2, 3) were aligned and concatenated using MEGA7 [26], and phylogenetic trees were constructed using the Neighbour-Joining method with model selection (Kimura 2-parameter method + Gamma distribution) determined by MEGA7 [27]. All trees were evaluated using 1000 nonparametric bootstraps.

**Table 2:** The accession numbers of sequences used in this study

Sample	*cox*1	16S	ITS1-5.8S-ITS2
*P. trivolvis*	AY227371.1	AY030234.1	AY030403.1
*B. glabrata* isolate VG3	JQ886409.1		
*B. glabrata* 1			AY030377.1
*B. glabrata*		AY198095.1	
*B. glabrata* 2			AY030376.1
*B. glabrata* 3			AY030375.1
*B. glabrata* 4			AY030374.1
*B. straminea* strain 1107	AF199085.1		
*B. straminea-*Brazil 2		AY030213.1	AY030381.1
*B. straminea* LBMSU431	KF926118.1		
*B. straminea-*Brazil 1		AY030214.1	AY030382.1
*B. prona*		AY030222.1	AY030391.1
*B. tenagophila*	AF199089.1	AY030220.1	AY030387.1
*B. amazonica*		AY030217.1	AY030385.1
*B. pfeifferi*	AF199105.1	AY030194.1	AY030364.1
*B. sudanica*	AF199109.1	DQ084861.1	AY030369.1
*B. camerunensis*	DQ084827.1	AY030198.1	AY030367.1
*B. kuhniana* Colombia		AY030211.1	
*B. kuhniana* SZX1	KJ137287.1	KJ137293.1	
*B. kuhniana*			AY030380.1
*B. kuhniana* Venezuela		AY030212.1

**Table 3:** The accession numbers of the sequences generated in this study

Sample	*cox*1	16S	ITS1-5.8S-ITS2
LW1	KY697197	KY697220	
LW2	KY697198	KY697221	KY684232
LW3	KY697199	KY697222	
HKL2	KY697200	KY697223	KY684234
HKL3	KY697201	KY697224	
HKL10	KY697202	KY697225	
PCL1	KY697203	KY697226	
PCL2	KY697204	KY697227	KY684238
PCL3	KY697205	KY697228	KY684239
SSH1	KY697206	KY697229	
SSH2	KY697207	KY697230	KY684241
SSH3	KY697208	KY697231	
NgT1	KY697209	KY697232	KY684243
NgT2	KY697210	KY697233	KY684244
NgT3	KY697211	KY697234	KY684245
SB1	KY697212	KY697235	
SB3	KY697213		
SG1	KY697214	KY697236	KY684246
SG3	KY697215		KY684247
SG4	KY697216	KY697238	KY684248
JP11	KY697217	KY697239	KY684249
JP12	KY697218	KY697240	KY684250
JP13	KY697219	KY697241	KY684251
DP2	KY697246	KY697245	KY690168
DP1	KY697247	KY697244	KY690167
DG11	KY697248	KY697242	KY690165
DG12	KY697249	KY697243	KY690166
ZD2	KY697250		
ZD1	KY697251		
LD4	KY697252		
LD5	KY697253		
LD6	KY697254		

### Detection of *S. mansoni* by both biopsy and molecular analyses

Field-collected snails were dissected under the microscope to look for any evidence of *S. mansoni*. In addition, a more reliable and sensitive molecular method specifically targeting the *S. mansoni* 16S rDNA sequence was employed. The following PCR temperature profile was used: an initial cycle of 5 min denaturation at 94 °C, followed by 40 iterations of 50s at 94 °C, 50 s at 55 °C and 50 s at 72 °C, and ended with 10 min extension at 72 °C. Gel electrophoreses were performed to confirm successful amplification of the genomic fragments with expected target size. PCR products were purified using the QIAquick PCR Product Purification Kit (Qiagen). Sequences of purified PCR products were obtained from both directions using the same primer pair for PCR by cycle sequencing in a commercial laboratory (BGI Tech Solutions (Hong Kong) Co., Ltd.).

## Results

### The distribution of *Biomphalaria straminea* in Hong Kong

**Fig. 1:**
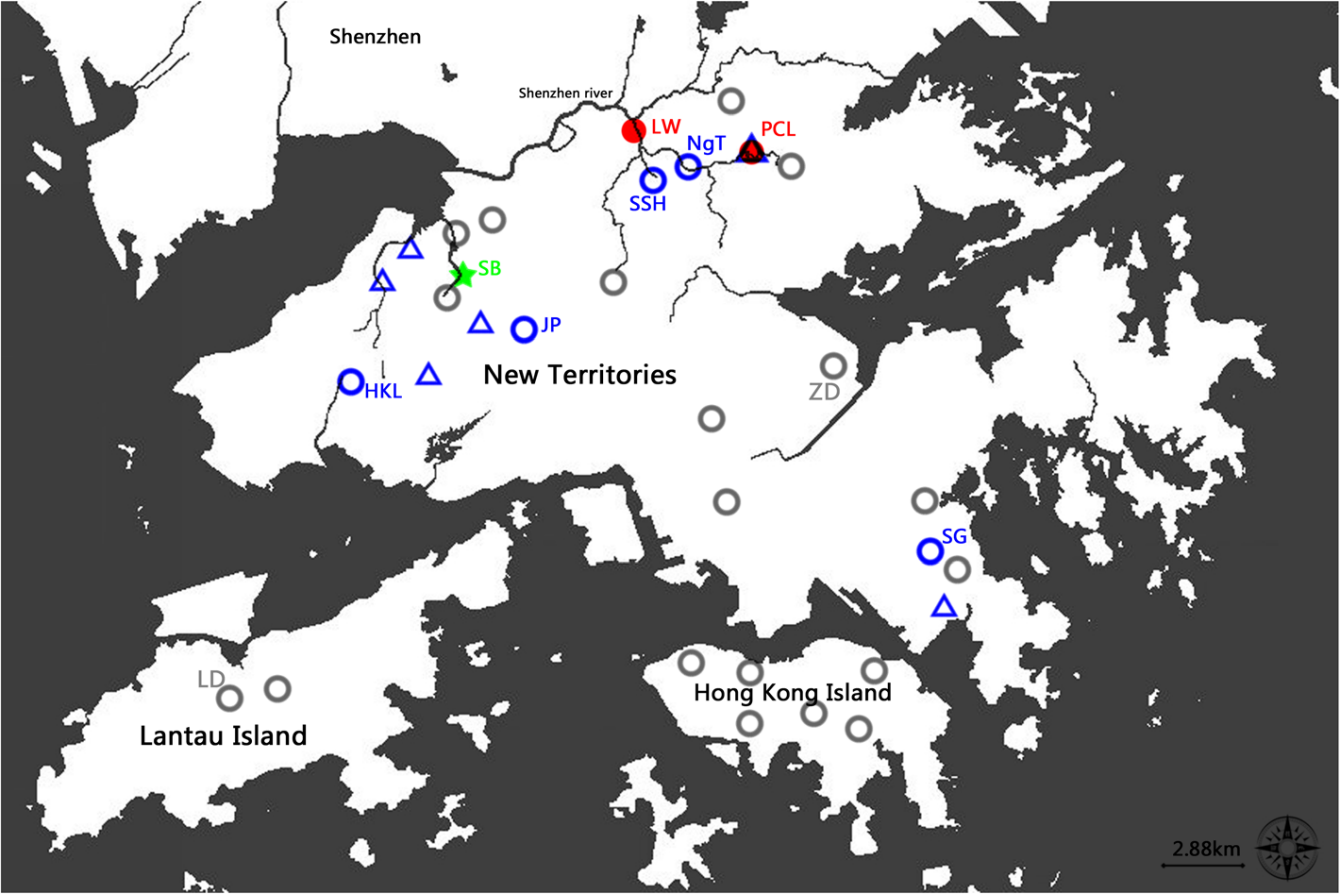
The geographical distribution of *B. straminea* in Hong Kong. The grey circles denote sites where *B. straminea* was absent; ZD and LD (marked in grey) represent sites containing snails with morphological resemblance to *B. straminea*, which were not *B. straminea* as suggested by the molecular data (i.e. false positive sites). The blue circles represent the *B. straminea*-positive sites whereas only black shell snails were found. The red dots represent *B. straminea*-positive sites with coexistence of red- and black- shelled snails. The green pentacle represents a site with only dead snails and shells found (proven to be *B. straminea* by molecular data). The blue triangles represent sites containing *B. straminea* reported in the 1980s [18]. Note that the site PCL contains *B. straminea* in both the present and previous studies

Taking into account the previous reports of *B. straminea* in Hong Kong back in the 1970s, a total of 27 freshwater locations in Hong Kong including 19 in the New Territories, 2 on Lantau Island and 6 on Hong Kong Island were visited between July 2016 and January 2017 (Table 1). Given that snails were collected based on morphological appearance, false positives were identified in sites such as the Tung Chung River and The Chinese University of Hong Kong on the basis of their *cox*1 sequences (see next section). Detailed anatomical and molecular analyses confidently indicated 8 positive sites containing *B. straminea*; these were widespread in the New Territories (NT) (i.e. 5 in northern NT close to the border to mainland China, 1 in south-western NT, 1 in central NT, and 1 in south-eastern NT) (Fig. 1).

**Fig. 2:**
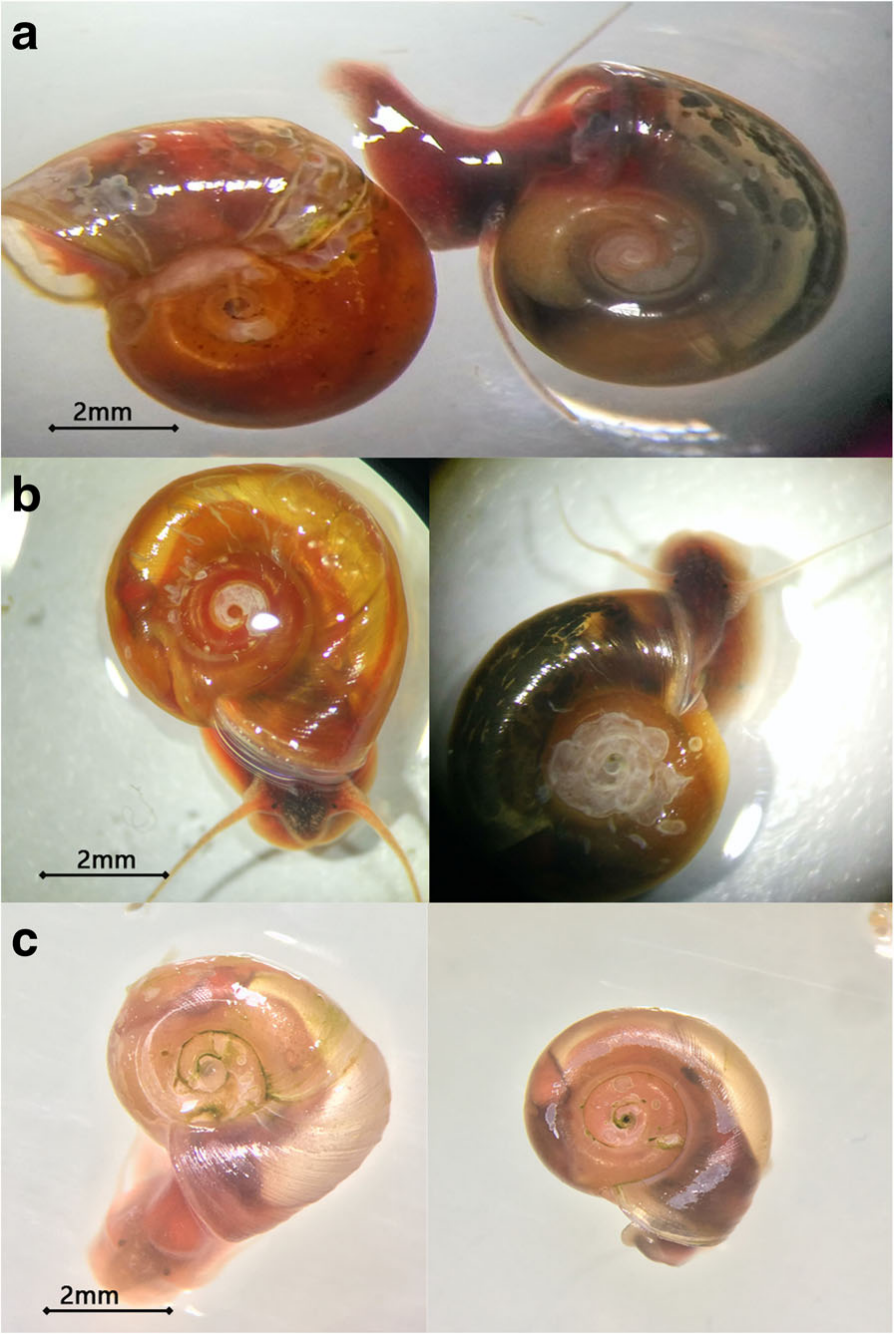
**a**, **b** Pictures of *B. straminea* snails captured in the field with red- and black-coloured shells. **c** Picture of *B. straminea* snails maintained in the lab with red-coloured shells. Note that the black eyespots are present in *B. straminea* with both red- and black-coloured shells

Black-coloured shell *B. straminea* were identified for all these 8 positive sites (Fig. 2); while red-coloured shell *B. straminea* were also found to coexist in certain locations, including the Lo Wu Border (LW) (i.e. the border between Hong Kong and mainland China) and Ping Che Road (PCL). In some locations, such as the one in Shan Pui River 1 (SB), only dead snails and empty shells could be found.

**Fig. 3:**
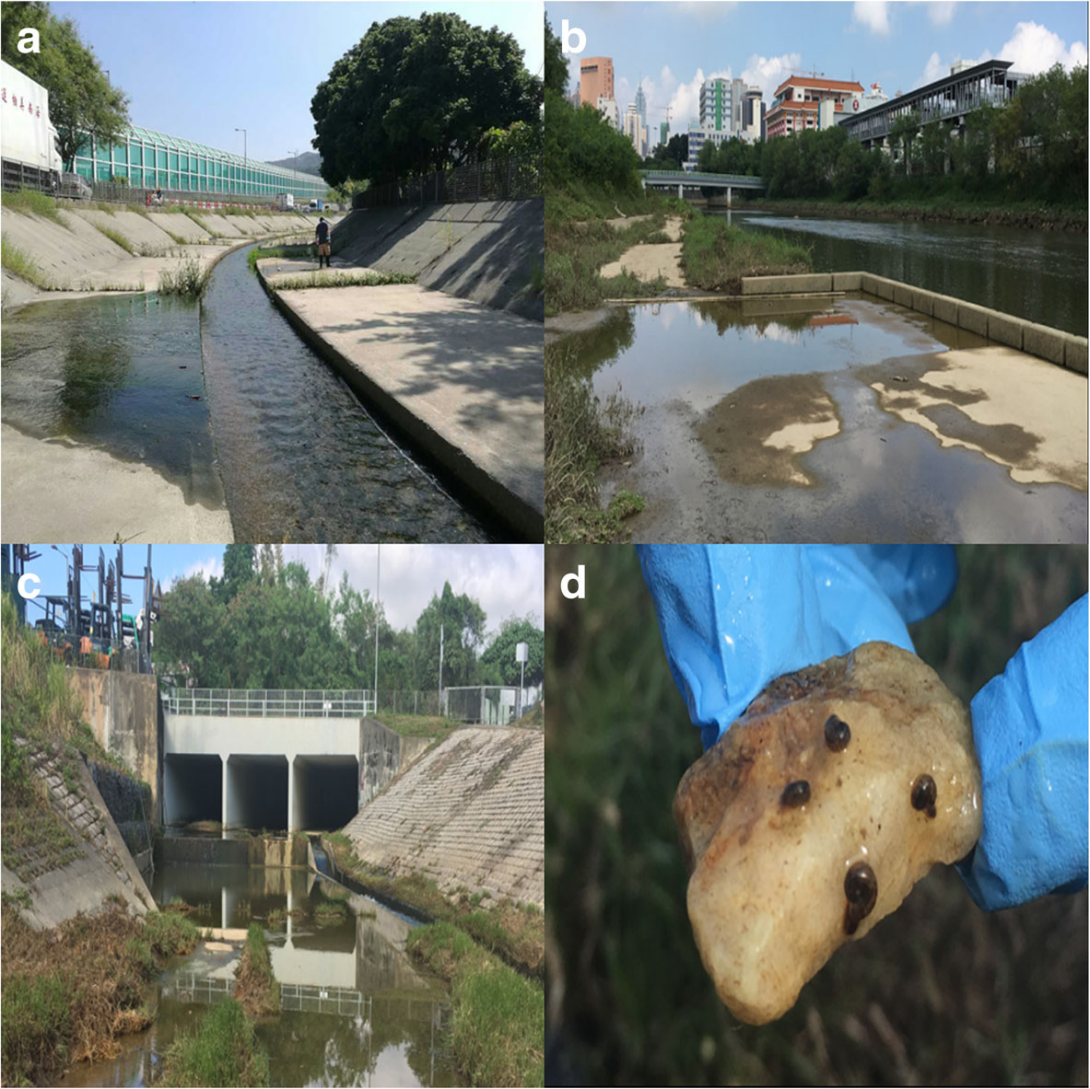
Pictures representing the typical habitats of snails *B. straminea* identified in Hong Kong. **a** Ditch in the Fu Hang Road (HKL). **b** River in the Lo Wu Border (LW). **c** Drainage exit in Ping Che Road (PCL). **d** Four *B. straminea* attached on a small rock collected in the field

Snails collected from in the field were cultured and allowed to breed in the laboratory (Fig. 3). Preliminary observations showed that adults with red-coloured shells caught from the wild produced offspring with red shells, while the first-generation offspring of black-shelled snails were either all with black shells or a mixture of individuals with black or red shells. Black eyespots were observed for both red-shelled and black-shelled *B. straminea* (Fig. 2b, c). Although a more detailed and mass-scale breeding exercise will be required to determine if this phenotype is controlled by a single allele, these observations supported the hypothesis that the red-shelled and black-shelled *B. straminea* do crossbreed in nature.

### Species identification of *B. straminea*

**Fig. 4:**
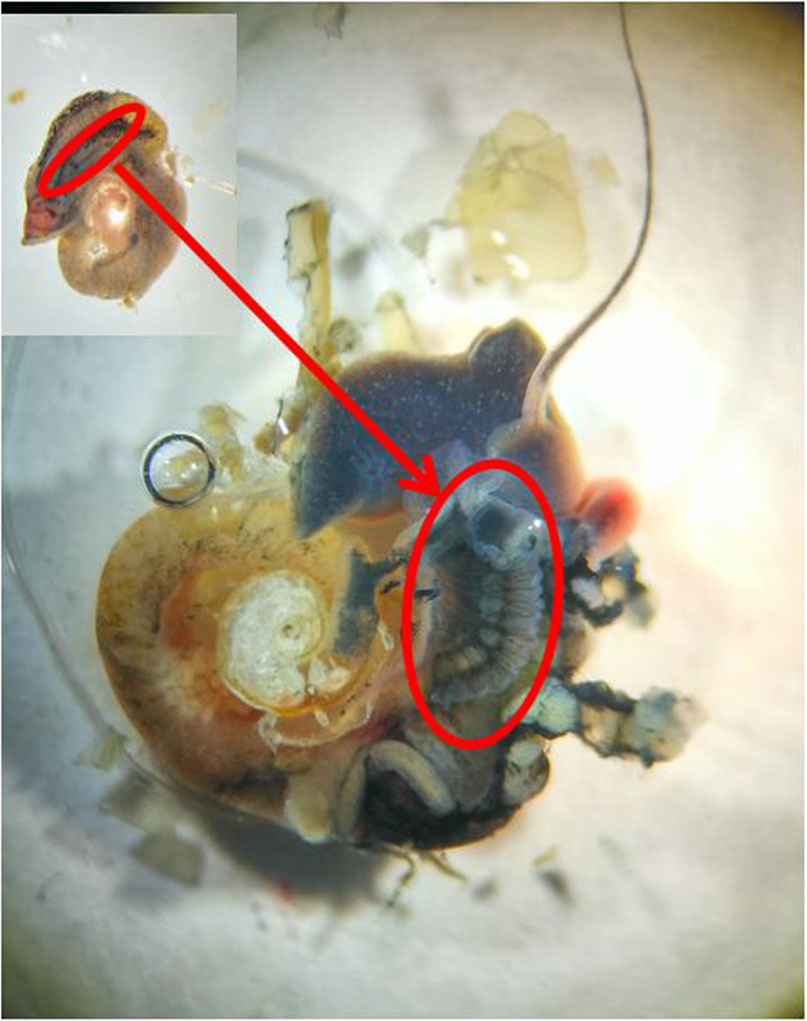
Picture of prostate diverticula in *B. straminea*. Tissue from the elliptic area (inset, circled in red) was cut open and the prostate diverticula (circled in red) were counted

**Fig. 5:**
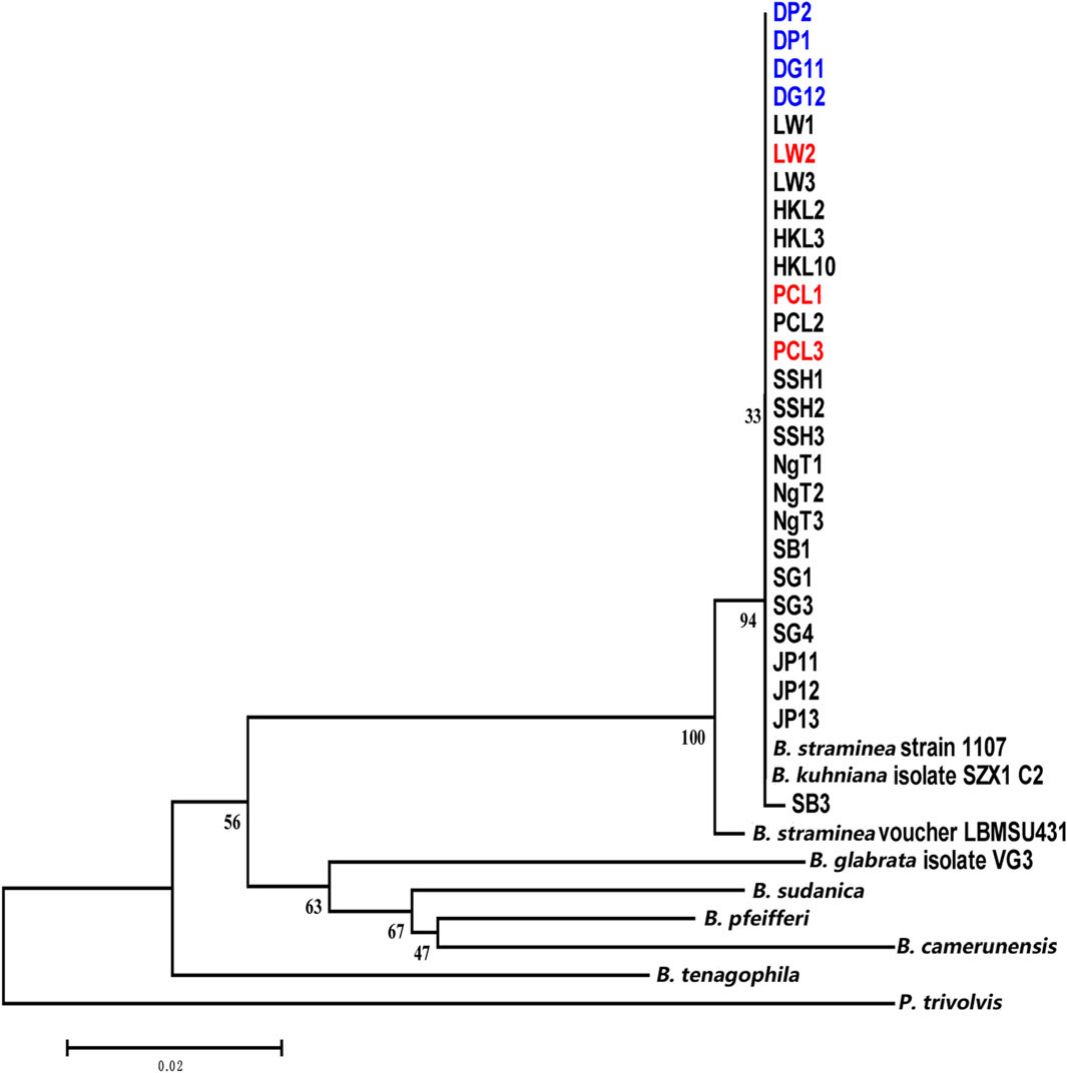
Neighbour-joining tree constructed based on K2P + G model for *cox*1 sequences (549 nt) of *B. straminea* samples collected from different sites in Hong Kong. The abbreviated names and numbers denote the samples collected from the sites listed in Table 1. Red colour represents *B. straminea* with red-coloured shells. DP1, DP2, DG11 and DG12 (marked in blue) represent *B. straminea* collected in cities outside Hong Kong (Shenzhen (DP) and DongGuan (DG) in the mainland China

Both molecular analysis on cytochrome *c* oxidase subunit 1 (*cox*1) sequences and anatomical analysis on prostate diverticula (Fig. 4) were applied to determine whether the specimens were bona fide *B. straminea* as previously described [22, 23]. As mentioned in the previous section, except for the snails collected from sites at the Tung Chung River (Lantau Island) and the Chinese University of Hong Kong (New Territories), all *cox*1 sequences proved to be similar to the *Biomphalaria straminea cox*1 sequence deposited on NCBI (GenBank: AF199085.1) (Fig. 5). For the other species, BlastN searches on the NCBI database revealed that one of the three samples collected at the Tung Chung River had 93% sequence similarity to *Hippeutis cantori* (GenBank: KC135955.1), while the two other samples collected from Tung Chung River and two samples collected from the Chinese University of Hong Kong all showed 99% sequence similarity to *Gyraulus* sp. (GenBank: KC495769.1). In addition, anatomical analyses were also carried out following the criteria of a previous study to differentiate *B. straminea* from *B. kuhniana* (a subspecies in *B. straminea*) [22], where the number of prostate diverticula in *B. straminea* are between 10 and 20 (average of 14.28, Table 4) and those in *B. kuhniana* are between 4 to 7. We examined the prostate diverticula of all these snails, except the ones collected from site SB which were decomposed (Table 4). All of these were found to fit the numbers for *B. straminea*, and hence, the combination of anatomical and molecular evidence indicates that they were all *B. straminea*.

**Table 4:** The number of prostate diverticula of the examined *B. straminea* captured from the field

Sample code	PDN	APDN
PCL1	14	13
PCL2	12	
PCL3	13	
LW1	16	14.67
LW2	14	
LW3	14	
SSH1	14	16.33
SSH2	17	
SSH3	18	
JP11	13	11.67
JP12	11	
JP13	11	
SG1	14	12.67
SG3	12	
SG4	12	
HKL2	14	12.67
HKL3	12	
HKL10	12	
NgT1	19	17.67
NgT2	18	
NgT3	16	
All		14.28

*Abbreviations*: *PDN* prostate diverticula number, *APDN* average prostate diverticula number

### Phylogenetic analysis of *B. straminea*

**Fig. 6:**
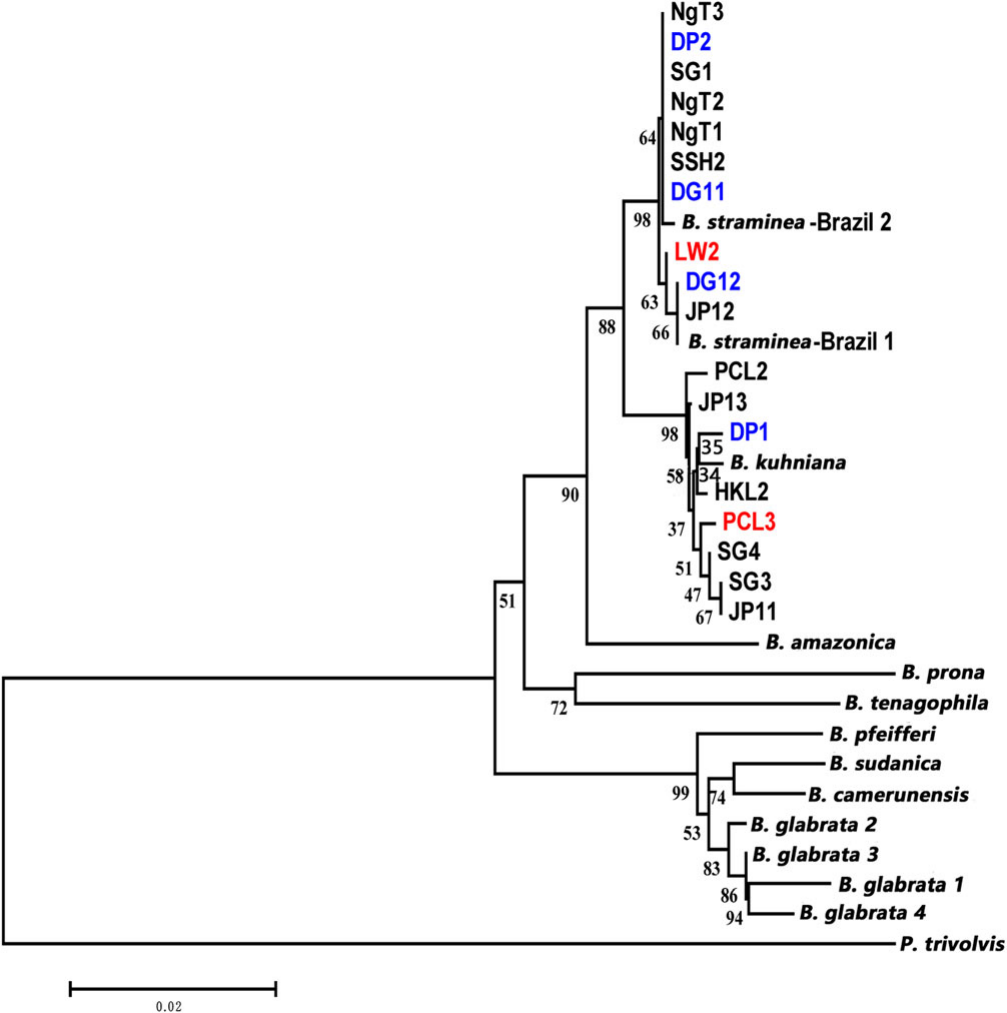
Neighbour-joining tree constructed based on K2P + G model for ITS1-5.8S-ITS2 sequences (914 nt) of *B. straminea* samples collected from different sites in Hong Kong. The abbreviated names and numbers denote the samples collected from the sites listed in Table 1. Red colour represents *B. straminea* with red-coloured shells. DP1, DP2, DG11 and DG12 (marked in blue) represent *B. straminea* collected in cities outside Hong Kong (Shenzhen (DP) and DongGuan (DG) in the mainland China

**Fig. 7:**
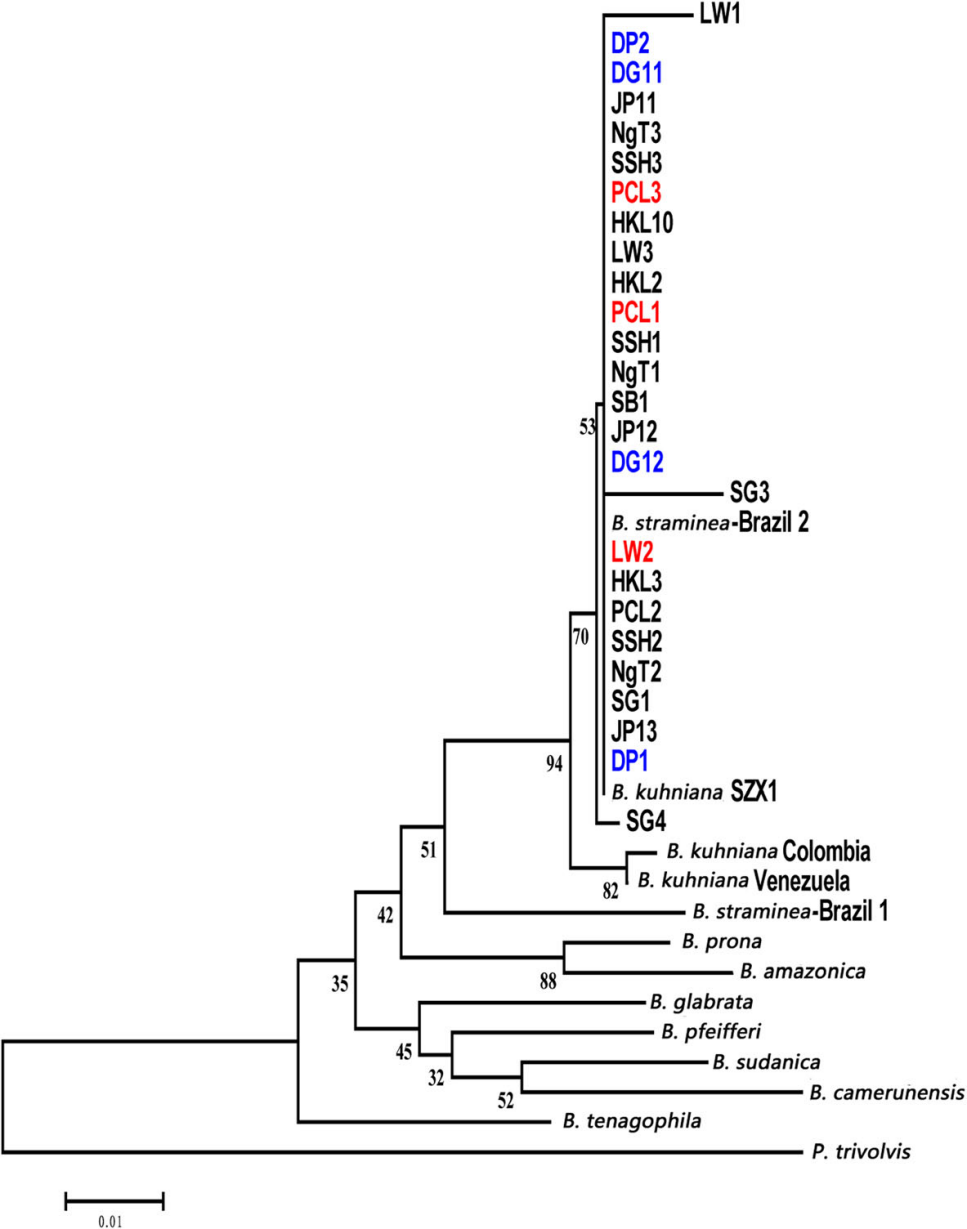
Neighbour-joining tree constructed based on K2P + G model for 16S rDNA sequences (343 nt) of *B. straminea* samples collected from different sites in Hong Kong. The abbreviated names and numbers denote the samples collected from the sites listed in Table 1. Red colour represents *B. straminea* with red-coloured shells. DP1, DP2, DG11 and DG12 (marked in blue) represent *B. straminea* collected in cities outside Hong Kong (Shenzhen (DP) and DongGuan (DG) in the mainland China

**Fig. 8:**
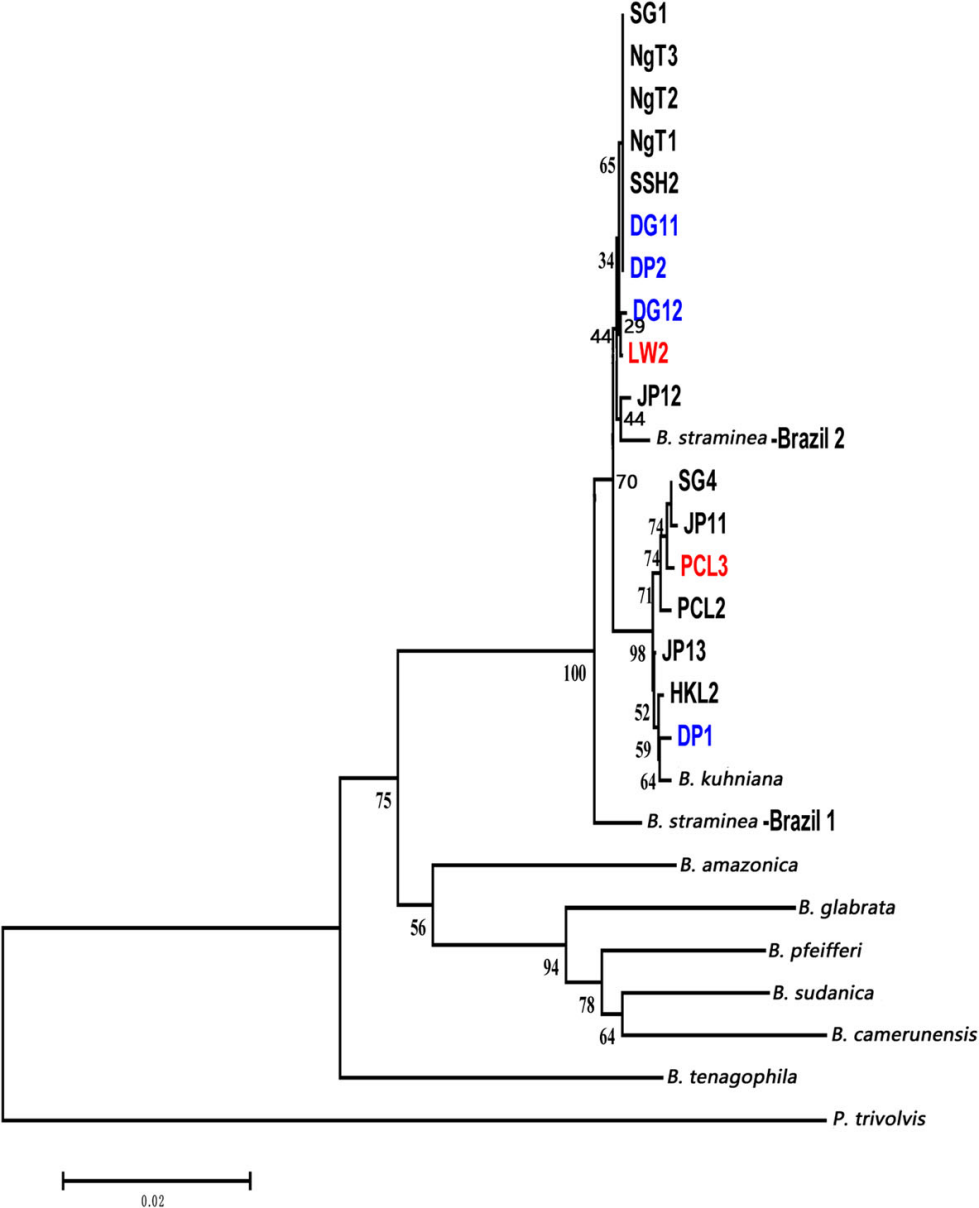
Neighbour-joining tree constructed based on K2P + G model for concatenated *cox*1, ITS1-5.8S-ITS2 and 16S rDNA sequences (1779 nt) of *B. straminea* samples collected from different sites in Hong Kong. The abbreviated names and numbers denote the samples collected from the sites listed in Table 1. Red colour represents *B. straminea* with red-coloured shells. DP1, DP2, DG11 and DG12 (marked in blue) represent *B. straminea* collected in cities outside Hong Kong (Shenzhen (DP) and DongGuan (DG) in the mainland China

*Cox*1, ITS1-5.8S-ITS2, and 16S rDNA were used as genetic markers in phylogenetic analyses of the *B. straminea* snails in Hong Kong. Maximum-likelihood trees based on individual markers demonstrated the *B. straminea* snails in Hong Kong were very similar to the Brazilian strain (Figs. 5, 6, 7; Additional file 1: Fig. S1, Additional file 2: Fig. S2 and Additional file 3: Fig. S3). Using the concatenated sequences from all three markers in constructing the phylogenetic trees resulted in the same result (Fig. 8; Additional file 4: Fig. S4), suggesting that the *B. straminea* snails in Hong Kong are likely derived from South America. The genetic distance tables are shown in Additional file 5: Tables S1, Additional file 6: Tables S2, Additional file 7: Tables S3 and Additional file 8: Tables S4).

### The infection status of *B. straminea* by *Schistosoma mansoni*

**Fig. 9:**
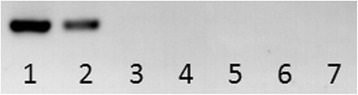
Example of PCR amplifying *S. mansoni* specific 16S rDNA sequence on gDNA extracted from: Lanes 1, 2: *S. mansoni* adult worms (as positive controls); Lane 3: field-captured *B. straminea* with a black-coloured shell; Lane 4: field captured *B. straminea* with a red-coloured shell; Lane 5: laboratory cultured *B. straminea* with a black-coloured shell; Lane 6: laboratory cultured *B. straminea* with a red-coloured shell; Lane 7: negative control. All 87 tested individuals were found to be negative for *S. mansoni* infection

Both the biopsy and molecular analyses using PCR primers specific for the *S. mansoni* 16S rDNA on *B. straminea* DNA gave negative results. A total of 87 individual snails were tested alongside the positive control which comprised DNA extracted from *S. mansoni*. Only the positive control was found to have a DNA fragment of 256 bp amplified when examined under the agarose gel, and all 87 samples tested were found to be negative (Fig. 9).

## Discussion

### Existence of *B. straminea* in contemporary Hong Kong

This study investigated the geographical distribution of *Biomphalaria straminea* in Hong Kong, its current infection status and revealed the phylogenetic relationships with those found in other parts of the world. The value of this study lies in determining the current distribution of these snails, which have the potential to transmit *S. mansoni* in contemporary Hong Kong. It is more than 30 years since the previous studies, and thus it is essential to evaluate the potential risk of the co-existence of *B. straminea* and the introduction of infection, i.e. infected individuals with *S. mansoni*, resulting in triggering the transmission cycle in the places concerned.

During this study carried out between July 2016 and January 2017, *B. straminea* was found in different districts in the New Territories, including the border between Hong Kong and mainland China. These districts include habitats which were not reported 30 years ago. Given there has been no systematic survey of snails in these districts in previous decades, and the New Territories have also been rapidly developed, it is not possible to determine whether the current population of *B. straminea* has been spreading and expanding in the last 30 years. On the other hand, highly urbanized and isolated areas, such as those in Hong Kong Island, where no signs of *B. straminea* could be detected, suggest a potential influence of urbanization on the snail’s habitat and distribution.

In the Shan Pui River 1 **(**SB), which is located near the estuary, only dead *B. straminea* or their empty shells could be found. If this observation was solely due to the influx of seawater, this would be indicative that *B. straminea* could not survive in brackish water, despite a previous report that concluded that *B. straminea* was more tolerant to both salinity and lack of freshwater than other *Biomphalaria* species [21].

### Red- and black-shelled *B. straminea* in Hong Kong

The identification of two shell colours of *B. straminea* contrasts with the previous field investigations in Hong Kong; either the colour variants were missed in the earlier studies or a new phenotype has evolved in the last 30 years [14, 15, 28–30]. A previous pigmentation study supported the existence of *B. straminea* with both shell colours [31]. Pigmentation effect has been better studied in another *Biomphalaria* species, *B. glabrata*, and temperature is one of the factors determining melanic and albino variants [32–34]. Nevertheless, given that black eyespots were observed in red shelled *B. straminea* (Fig. 2), the genetic mutation responsible is likely to be different from true albinos.

### Genetic structure of *B. straminea* in Hong Kong

A recent survey on the distribution of *Biomphalaria* spp. in mainland China suggested the presence of two genetically close species, *B. straminea* and *B. kuhniana*, in Guangdong Province (southern part of mainland China and connected to the border of Hong Kong) [15]. Given the two species have different abilities to transmit *S. mansoni* (*B. kuhniana* is thought to be incapable), anatomical analysis was first employed, revealing the average number of prostate diverticula to be 14.28 (Table 4), indicating that the snails were *B. straminea* and not *B. kuhniana*. The phylogenetic relationships of *B. straminea* snails collected in the present study were assessed using a combination of 16S ribosomal DNA, 18S ribosomal DNA + internal transcribed spacer 1 + 5.8S ribosomal DNA + internal transcribed spacer 2 + 28S ribosomal DNA and *cox*1 region markers [35–37] and the results clearly showed that the snails collected in Hong Kong are genetically indistinguishable, similar to those in mainland China, and phylogenetically close to those from Brazil in South America (Fig. 8). All these data suggest that the *B. straminea* snails in Hong Kong and maybe also mainland China are likely derived from South America.

### Potential risk of schistosomiasis?

Recent studies have shown that *Biomphalaria* snails including *B. straminea* in the Guangdong Province, southern part of mainland China closest to the border of Hong Kong, had been expanding their range from isolated sites in Shenzhen, not only to all districts in Shenzhen including Dongguan and Huizhou cities, but to the entrance of the East Branch of Pearl River [28, 29]. It has also been predicted that *B. straminea* will spread to Pearl River Delta areas in Guangdong, southern parts of Guangxi and Fujian provinces, as well as the North of Taiwan [30]. These data suggest that the *B. straminea* situation should be closely monitored.

Ever since the first report of identification of *B. straminea* in Hong Kong back in the 1970s, there have always been concerns about *S. mansoni* infection breakouts southern China [14–20, 28–30]. In the north-east of Brazil, the prevalence of *S. mansoni* infection in *B. straminea* ranges from 11 to 24% [38, 39], while artificial exposures of *S. mansoni* to *B. straminea* in the laboratory have shown low infectivity, with prevalence ranging from 1.2 to 1.6% depending on the strain of miracidia being tested [40]. Close to the border between Hong Kong and mainland China, in the city of Shenzhen, a recent study reported that the black-coloured *B. straminea* collected in the field were resistant to *S. mansoni* infection [41]. All 87 individuals collected from the field and analysed in this study were found to be negative for *S. mansoni* but the susceptibility of cultured snails to infection in the laboratory with *S. mansoni* remains to be tested.

In 2016, there were more than 56 million visitors worldwide travelling to Hong Kong including those from *S. mansoni*-endemic countries. The number of trips for people travelling between Hong Kong and mainland China was also more than 77 million per year. The potential risk of introducing and spreading schistosomiasis exists. Yet, without knowing the biology of the vector *B. straminea*, such as its transmission ability in Hong Kong and mainland China when compared to those reported in Brazil for instance, it is difficult to determine the actual risk (i.e. high, medium, low, or negligible). Nevertheless, given the increasing range of *B. straminea*, an updated strategy is required to minimize the risk of spreading schistosomiasis; such a strategy could include surveillance of potentially infected migrants or overseas workers, regulating the import of freshwater plants, and snail control using molluscicides in potential transmission sites.

## Conclusions

This study revealed the presence of *B. straminea* in different districts in the New Territories of Hong Kong, including places close to the border connecting to mainland China. None of the *B. straminea* investigated in this study were found to be infected with *S. mansoni*. The Hong Kong *B. straminea* are genetically indistinguishable, based on the chosen markers, and resemble those from South America.

## Additional files

Additional file 1: Figure S1. Maximum-likelihood tree constructed based on Hasegawa-Kishino-Yano model + G model for *cox*1 sequences (549 nt) of *B. straminea* samples collected from different sites in Hong Kong. The abbreviated names and numbers denote the samples collected from the sites listed in Table 1. Red colour represents *B. straminea* with red-coloured shells. DP1, DP2, DG11 and DG12 (marked in blue) represent *B. straminea* collected in cities outside Hong Kong (Shenzhen (DP) and DongGuan (DG) in the mainland China. (PDF 184 kb)

Additional file 2: Figure S2. Maximum-likelihood tree constructed based on K2P + G model for ITS1-5.8S-ITS2 sequences (914 nt) of *B. straminea* samples collected from different sites in Hong Kong. Abbreviations are as in Figure S1. (PDF 186 kb)

Additional file 3: Figure S3. Maximum-likelihood tree constructed based on Tamura 3-parameter + G model for 16S rDNA sequences (343 nt) of *B. straminea* samples collected from different sites in Hong Kong. Abbreviations are as in Figure S1. (PDF 185 kb)

Additional file 4: Figure S4. Maximum-likelihood tree constructed based on General Time Reversible + G + I model for concatenated *cox*1, ITS1-5.8S-ITS2 and 16S rDNA sequences (1779 nt) of *B. straminea* samples collected from different sites in Hong Kong. Abbreviations are as in Figure S1. (PDF 185 kb)

Additional file 5: Table S1. Genetic distance table for *cox*1 sequences used in Fig. 5. (XLS 39 kb)

Additional file 6: Table S2. Genetic distance table for ITS1-5.8S-ITS2 sequences used in Fig. 6. (XLS 42 kb)

Additional file 7: Table S3. Genetic distance table for 16S rDNA sequences used in Fig. 7. (XLS 42 kb)

Additional file 8: Table S4. Genetic distance table for concatenated *cox*1, ITS1-5.8S-ITS2 and 16S rDNA sequences used in Fig. 8. (XLS 37 kb)

## Abbreviations

APDN: Average diverticula number
CDC: Guangdong Provincial Center for Disease Control and Prevention
*cox*1: cytochrome *c* oxidase subunit 1
ITS: Internal transcribed spacer
PCR: Polymerase chain reaction
PDN: Prostate diverticula number
rDNA: ribosomal DNA
