# Supplementary figures and images for: Distribution and current infection status of Biomphalaria straminea in Hong Kong
Source: Parasit Vectors. 2017 Jul 25;10:351. doi: 10.1186/s13071-017-2285-3 (PMC5526268; doi:10.1186/s13071-017-2285-3)

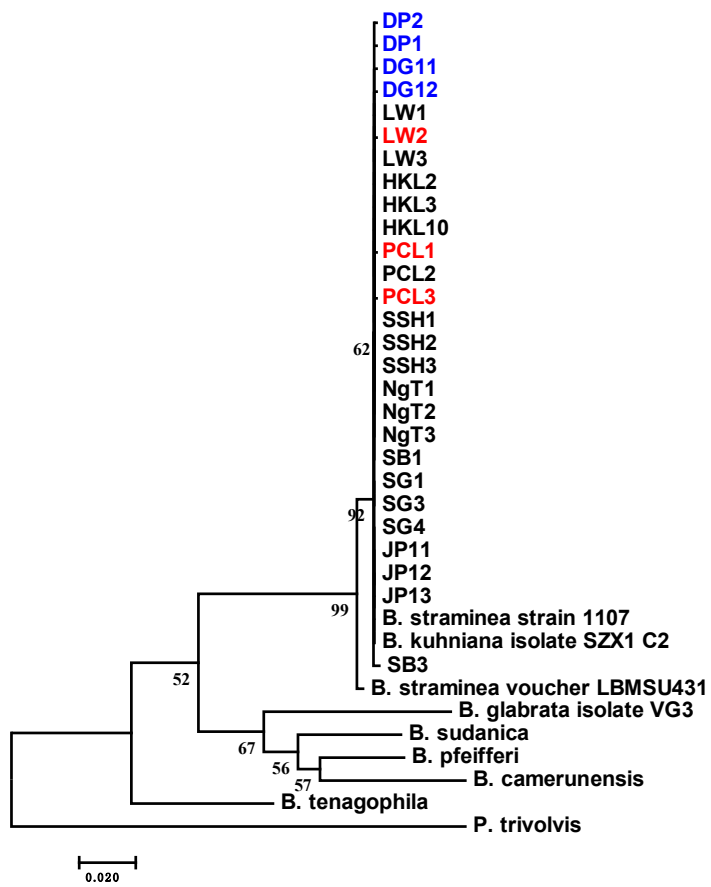

Supplement: Supplementary file 1 — Maximum-likelihood tree constructed based on Hasegawa-Kishino-Yano model + G model for cox1 sequences (549 nt) of B. straminea samples collected from different sites in Hong Kong. The abbreviated names and numbers denote the samples collected from the sites listed in Table 1. Red colour represents B. straminea with red-coloured shells. DP1, DP2, DG11 and DG12 (marked in blue) represent B. straminea collected in cities outside Hong Kong (Shenzhen (DP) and DongGuan (DG) in the mainland China. (PDF 184 kb) [file 13071_2017_2285_MOESM1_ESM.pdf]

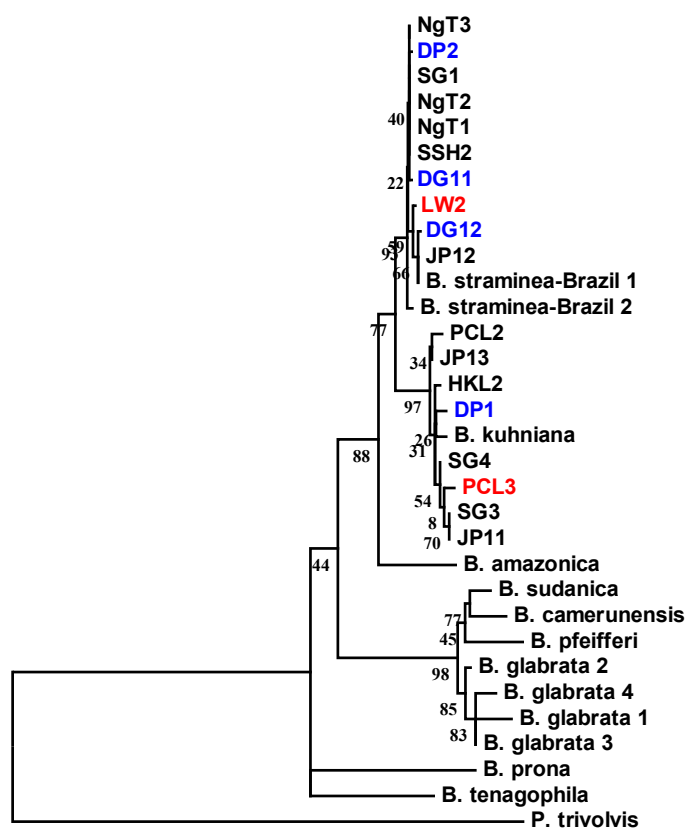

0.02

Supplement: Supplementary file 2 — Maximum-likelihood tree constructed based on K2P + G model for ITS1-5.8S-ITS2 sequences (914 nt) of B. straminea samples collected from different sites in Hong Kong. Abbreviations are as in Figure S1. (PDF 186 kb) [file 13071_2017_2285_MOESM2_ESM.pdf]

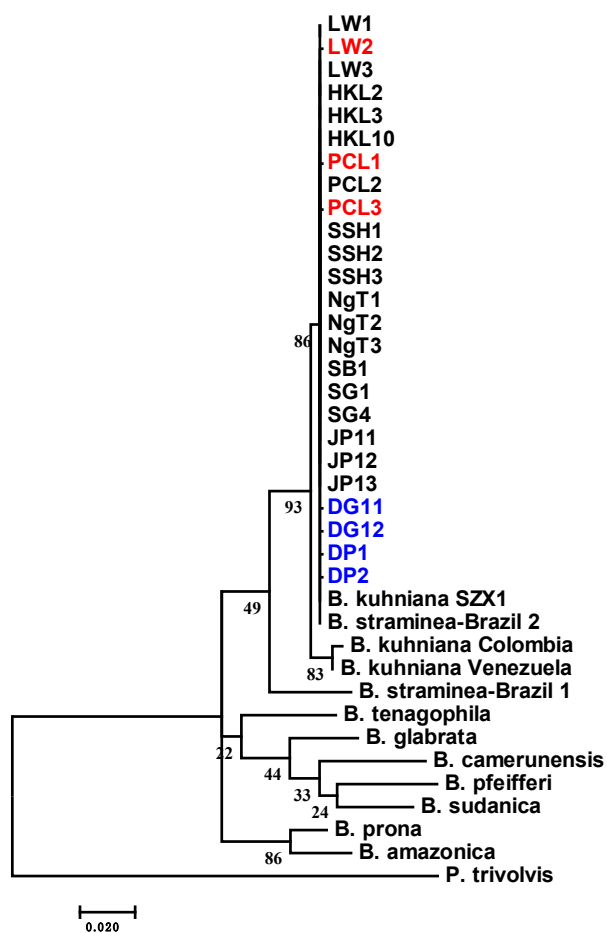

Supplement: Supplementary file 3 — Maximum-likelihood tree constructed based on Tamura 3-parameter + G model for 16S rDNA sequences (343 nt) of B. straminea samples collected from different sites in Hong Kong. Abbreviations are as in Figure S1. (PDF 185 kb) [file 13071_2017_2285_MOESM3_ESM.pdf]

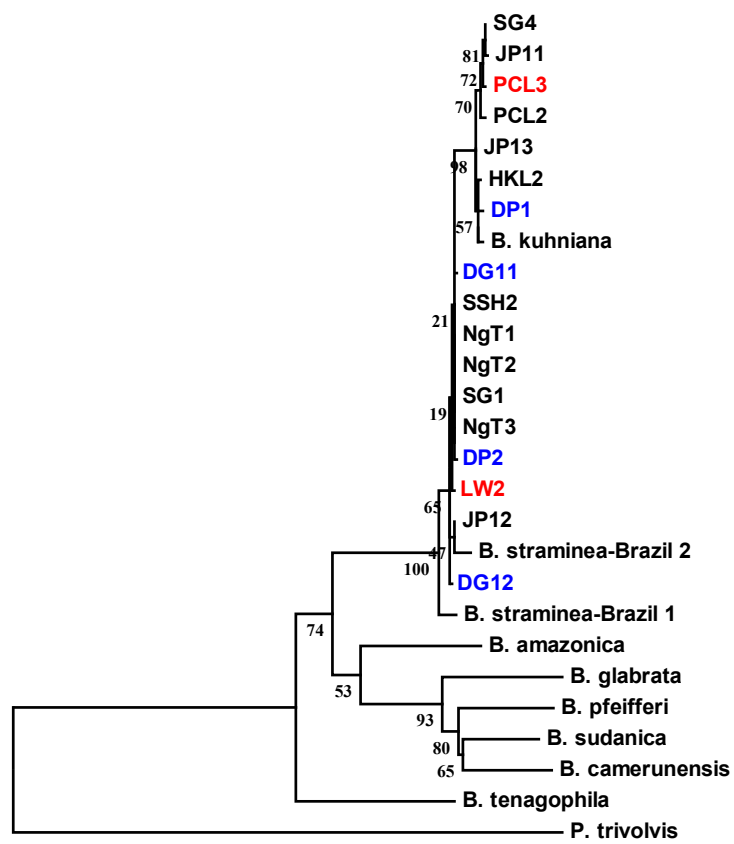

Supplement: Supplementary file 4 — Maximum-likelihood tree constructed based on General Time Reversible + G + I model for concatenated cox1, ITS1-5.8S-ITS2 and 16S rDNA sequences (1779 nt) of B. straminea samples collected from different sites in Hong Kong. Abbreviations are as in Figure S1. (PDF 185 kb) [file 13071_2017_2285_MOESM4_ESM.pdf]
